# Supplementary material for: Time dependent response of daunorubicin on cytotoxicity, cell cycle and DNA repair in acute lymphoblastic leukaemia
Source: BMC Cancer. 2019 Feb 27;19:179. doi: 10.1186/s12885-019-5377-y (PMC6391779; doi:10.1186/s12885-019-5377-y)

Supporting material for

Time dependent response of daunorubicin on cytotoxicity, cell cycle and DNA repair in Acute Lymphoblastic Leukaemia

Hussain Mubarak Al-Aamri[1], Heng Ku[1], Helen R Irving[1*], Joseph Tucci[1], Terri Meehan-Andrews[1] and Christopher Bradley[1]

[1]Department of Pharmacy and Applied Sciences, La Trobe Institute for Molecular Science (LIMS), La Trobe University, P.O. Box 199, Bendigo, Victoria, Australia

*Corresponding author

E-mail: h.irving@latrobe.edu.au (HRI)

Supporting Tables

Table S1. Primers used for PCR.

**Primer sets**	**Sequence**	**Expected product size**
00 Forward	5’ TTAACAATGAAGATGGTGCTC 3’	914 bp
00 Reverse	5’ CTTAAAGTATGTTGGCAGGT 3’	
0 Forward	5’ ACTAAACCAGAGGTAGCCAGAAG 3’	837 bp
0 Reverse	5’ AGCCAATCGCTTCTCAAACC 3’	
1 Forward	5’ GTCTGAGGGTTTGTGGCAACTG 3’	844 bp
1 Reverse	5’ CTGACCATCTGAGGTCTCCTACTTC 3’	
2 Forward	5’ GCCAGGCAGGAATCATTCAG 3’	1000 bp
2 Reverse	5’ AACCGGGCTAATGAGAGAAAT GC 3’	
3 Forward	5’ TAGCCACAAAGACTGGACATA G 3’	1129 bp
3 Reverse	5’ TTGCTGACGGAAGTGCAATG 3’	
4 Forward	5’ GTGGCTTAGGAGGAGCTTGG 3’	933 bp
4 Reverse	5’ GCTGTAGATAGGCCAGCATTG 3’	
5 Forward	5’ TTATCCTGTAGCCCTATCTGC 3’	1004 bp
5 Reverse	5’ TCACAACAAAGGGAGAAGCTACG 3’	
6 Forward	5’ AAAGGAGCTTCCTGGAGAAGAG 3’	964 bp
6 Reverse	5’ AGCAATGGACTTCACCTCATC 3’	
7 Forward	5’ CACCAGAATCTCAAGGAATCAC 3’	996 bp
7 Reverse	5’ AGCATCCCTTGTGTTCTCAG 3’	
8 Forward	5’ TGTTACGATGCCTTACGGAAGTTG 3’	832 bp
8 Reverse	5’ ATGTAACAGTAGCAGCCAAGGAC 3’	
9 Forward	5’ CTCTGTGTACTTCAGGCTCTATC 3’	918 bp
9 Reverse	5’ GCTTTCTAGGTTTGACCTCTTG 3’	
9.5 Forward	5’ CTTAATGATCTGCTTATCTGCTGC 3’	874 bp
9.5 Reverse	5’ TGGCTCCTTTCGGATGATGG 3’	
10 Forward	5’ CTTAATGATCTGCTTATCTGCTGC 3’	409 bp
10 Reverse	5’ AATCAGCTCCGTAAATAGCACC 3’	

Table S2. PCR conditions for the different primer sets.

Primer set	Initial denaturation (minutes/°*C)*	Denaturation (second/°*C)*	Annealing (second/°*C)*	Extension (minutes/°*C)*	Cycles	Final extension (minutes/°*C)*
1-5	95°*C for 10 minutes*	95°*C, 30 sec*	57°*C, 30 sec*	72°*C, 1 min*	30	*72°C, 5 min*
00,0, 6-9	95°*C for 10 minutes*	95°*C, 30 sec*	53°*C, 30 sec*	72°*C, 1 min*	30	*72°C, for 5 min*
9.5,10	95°*C for 10 minutes*	95°*C, 30 sec*	50°*C, 30 sec*	72°*C, 1 min*	30	*72°C, for 5 min*

Supporting Figures

Fig S1. Examples of histograms used for the ROS assay. Illustrative histograms obtained from (A) MOLT-4, (B) CCRF-CEM, and (C) SUP-B15 cell lines.

(A) MOLT-4

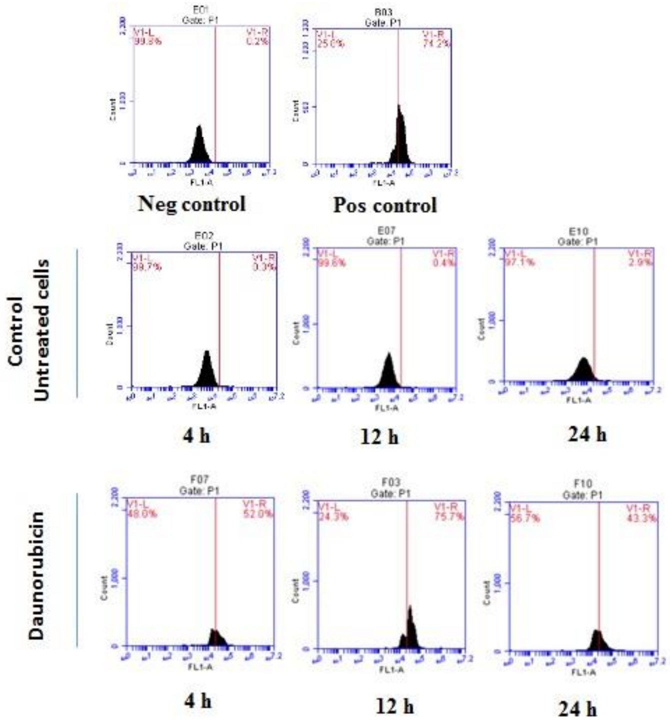

(B) CCRF-CEM

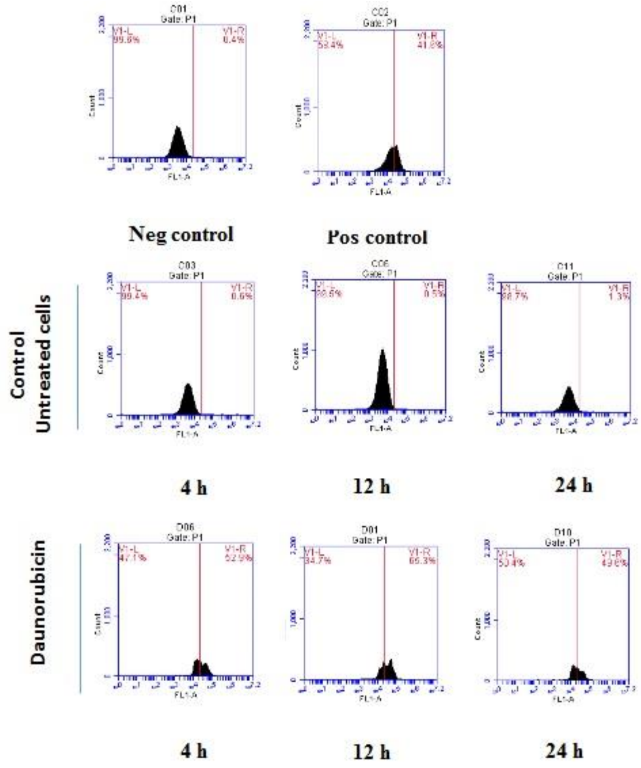

(C) SUP-B15

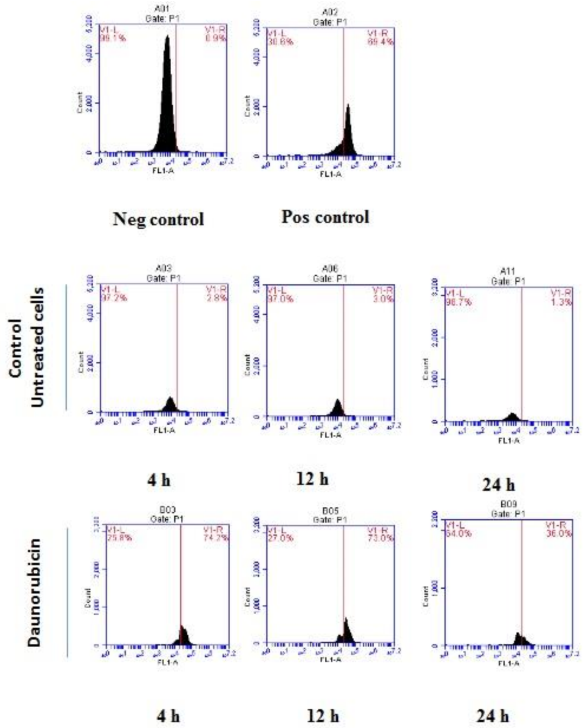
Neg control
Pos control
Control
Untreated cells
4 h
12 h
24 h
Daunorubicin
4 h
12 h
24 h

Fig S2. Examples of histograms used for the gamma H2AX assay. Illustrative histograms obtained from (A) MOLT-4, (B) CCRF-CEM, and (C) SUP-B15 cell lines

(A) MOLT-4

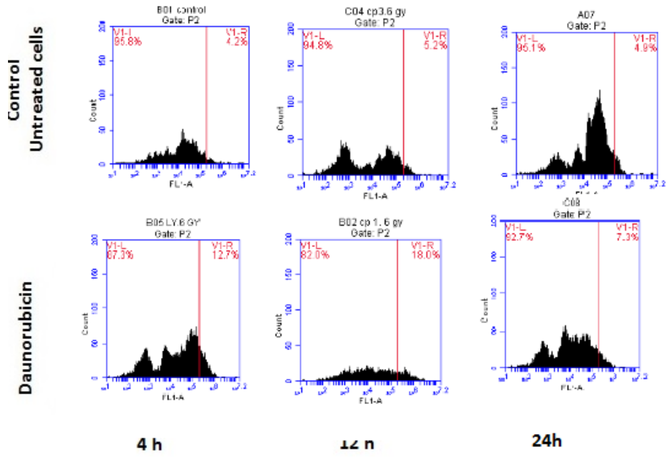

(B) CCRF-CEM

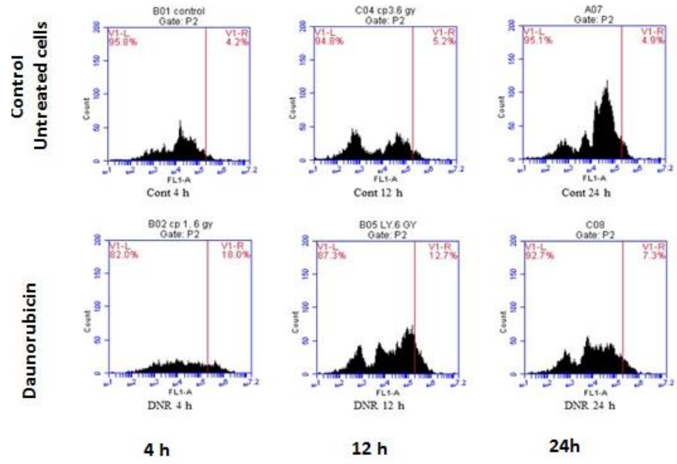

(C) SUP-B15

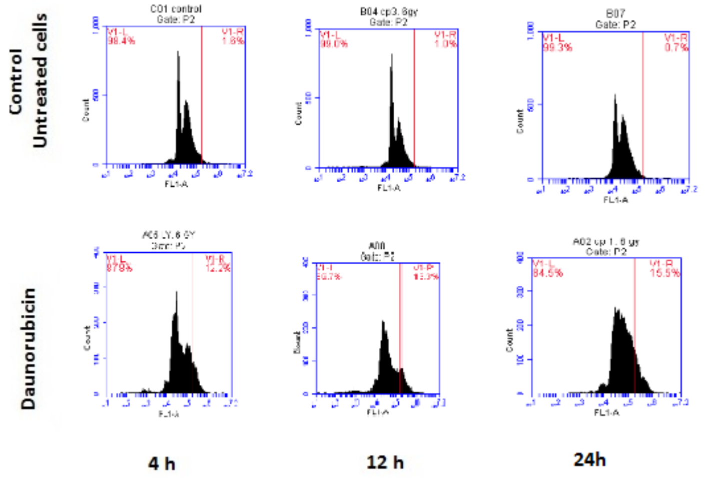

Supplement: Supplementary file 1 — Figure S1. Examples of histograms used for the ROS assay. Illustrative histograms obtained from (A) MOLT-4, (B) CCRF-CEM, and (C) SUP-B15 cell lines. Figure S2. Examples of histograms used for the gamma H2AX assay. Illustrative histograms obtained from (A) MOLT-4, (B) CCRF-CEM, and (C) SUP-B15 cell lines. Table S1. Primers used to amplify the ATM cDNA. Table S2. PCR conditions for the different primer sets to amplify ATM. (PDF 286 kb) [file 12885_2019_5377_MOESM1_ESM.pdf]
